# Mobile Phone Ownership, Health Apps, and Tablet Use in US Adults With a Self-Reported History of Hypertension: Cross-Sectional Study

**DOI:** 10.2196/12228

**Published:** 2019-01-14

**Authors:** Aisha T Langford, Craig A Solid, Ebony Scott, Meeki Lad, Eli Maayan, Stephen K Williams, Azizi A Seixas

**Affiliations:** 1 Division of Comparative Effectiveness and Decision Science Department of Population Health NYU School of Medicine New York, NY United States; 2 Solid Research Group, LLC St. Paul, MN United States; 3 The Langford Lab Department of Population Health NYU School of Medicine New York, NY United States; 4 Center for Healthful Behavior Change Department of Population Health NYU School of Medicine New York, NY United States

**Keywords:** smartphone, text messaging, health communication, ownership, goals, cell phone, telemedicine, hypertension, tablets, chronic disease

## Abstract

**Background:**

Mobile phone and tablet ownership have increased in the United States over the last decade, contributing to the growing use of mobile health (mHealth) interventions to help patients manage chronic health conditions like diabetes. However, few studies have characterized mobile device ownership and the presence of health-related apps on mobile devices in people with a self-reported history of hypertension.

**Objective:**

This study aimed to describe the prevalence of smartphone, tablet, and basic mobile phone ownership and the presence of health apps by sociodemographic factors and self-reported hypertension status (ie, history) in a nationally representative sample of US adults, and to describe whether mobile devices are associated with health goal achievement, medical decision making, and patient-provider communication.

**Methods:**

Data from 3285 respondents from the 2017 Health Information National Trends Survey were analyzed. Participants were asked if they owned a smartphone, tablet, or basic mobile phone and if they had health apps on a smartphone or tablet. Participants were also asked if their smartphones or tablets helped them achieve a health-related goal like losing weight, make a decision about how to treat an illness, or talk with their health care providers. Chi-square analyses were conducted to test for differences in mobile device ownership, health app presence, and app helpfulness by patient characteristics.

**Results:**

Approximately 1460 (37.6% weighted prevalence) participants reported a history of hypertension. Tablet and smartphone ownership were lower in participants with a history of hypertension than in those without a history of hypertension (55% vs 66%, *P*=.001, and 86% vs 68%, *P*<.001, respectively). Participants with a history of hypertension were more likely to own a basic mobile phone only as compared to those without a history of hypertension (16% vs 9%, *P*<.001). Among those with a history of hypertension exclusively, basic mobile phone, smartphone, and tablet ownership were associated with age and education, but not race or sex. Older adults were more likely to report having a basic mobile phone only, whereas those with higher education were more likely to report owning a tablet or smartphone. Compared to those without a history of hypertension, participants with a history of hypertension were less likely to have health-related apps on their smartphones or tablets (45% vs 30%, *P*<.001) and report that mobile devices helped them achieve a health-related goal (72% vs 63%, *P*=.01).

**Conclusions:**

Despite the increasing use of smartphones, tablets, and health-related apps, these tools are used less among people with a self-reported history of hypertension. To reach the widest cross-section of patients, a mix of novel mHealth interventions and traditional health communication strategies (eg, print, web based, and in person) are needed to support the diverse needs of people with a history of hypertension.

## Introduction

In recent years, mobile health (mHealth) interventions have been proposed as promising strategies for delivering health interventions to people with chronic health conditions [1,2]. For example, mHealth interventions for type 2 diabetes account for a sizable proportion of published articles [1-5]. However, the literature on mHealth interventions for cardiovascular disease generally and hypertension specifically is less robust [6-8]. This gap in the literature is notable, given that heart disease is the primary cause of death among adults in the United States [9,10] and hypertension is a major risk factor for heart disease [10-13]. Recent reports estimate that the proportion of US adults with hypertension is approximately 46% [13,14]. If people in the prehypertension range are considered, hypertension becomes a concern for more than half of US adults, thereby highlighting the need to initiate more mHealth approaches to help prevent or manage hypertension (eg, medication management and lifestyle change).

Broadly, mHealth is conceptualized as an area of electronic health (eHealth) that uses mobile technologies such as mobile phones and wireless devices for health research and healthcare delivery [15,16]. Examples of mHealth interventions include short message service text messaging, telephone-delivered interventions (eg with a nurse or health coach), Bluetooth-enabled pill boxes and fitness monitors, and health-related smartphone apps [17-20]. A report published by the Pew Research Center in 2015 noted that text messaging was the most widely used smartphone basic feature or app, with approximately 97% of smartphone owners reporting that they used text messaging at least once over the course of 1 week [21]. This finding was part of an “experience sampling” survey conducted by Pew Research Center in which smartphone owners were contacted twice a day for a week and queried about how they used their smartphone in the hour immediately before answering survey questions [21].

The use of mHealth interventions over time has largely mirrored the rapid growth in ownership of smartphones and other devices over the last two decades [2,22,23]. In 2018, approximately 95% of Americans owned a mobile phone of some kind [23]. Between 2010 and 2016, the Pew Research Center reported that smartphone ownership among Americans increased from 35% to 77% and tablet ownership increased from 3% to 51% [24]. Among adults who own basic mobile phones only, those aged ≥65 years comprise the largest proportion (40%) compared to all other age groups. With regard to race, the proportion of basic mobile phone ownership for white, black, and Hispanic people was 17%, 23%, and 20%, respectively [23]. With regard to mobile health apps, more than 300,000 apps are available to the general public to download from Google Play and the Apple Store [25]. Some early evidence suggests that apps are associated with behavior change intentions as well as actual behavior change related to diet, physical activity, weight loss, and smoking cessation [26,27]. However, few health-related apps are evidence based [28] and few are consistently used over time [27,29].

Despite the ubiquitous use of mobile phones in the United States and growing interest among researchers to develop mHealth interventions for people with chronic diseases, the data are still limited with regard to mobile phone ownership and the use of mHealth interventions among people with a self-reported history of hypertension. Thus, the objectives of this study were to answer four key questions:

1. Do people with a self-reported history of hypertension differ from those without a self-reported history of hypertension in terms of basic mobile phone, smartphone, and tablet ownership?
2. Among people with a self-reported history of hypertension exclusively, does mobile device ownership differ by age, gender, race/ethnicity, or education?
3. Are there differences between people with and those without a self-reported history of hypertension with regard to having health-related apps on their smartphones and tablets?
4. How do people with a self-reported history of hypertension differ from those without a self-reported history of hypertension with regard to the role that smartphones and tablets play in helping them achieve health-related goals, make medical decisions, or establish patient-provider communication?

Given the large number of US adults affected by hypertension [13] and growing interest in the promise of mHealth interventions, our findings may help inform future efforts to develop hypertension-focused mHealth interventions for patients in clinical settings and the general public.

## Methods

### Overview of the Health Information National Trends Survey

The Health Information National Trends Survey (HINTS) is a probability-based, nationally representative survey of US noninstitutionalized adults aged ≥18 years [30], sponsored by the US Department of Health and Human Services. HINTS has been administered approximately every 1-3 years since 2003, with the goal of collecting nationally representative data that track changes in health communication and information technology. The sample design for the 2017 HINTS 5, Cycle 1, consisted of a single-mode mail survey using the next-birthday method for respondent selection and comprised two stages. In the first stage, a stratified sample of addresses was selected from a file of residential addresses. In the second stage, one adult was selected within each sampled household. The sampling frame consisted of a database of addresses used by Marketing Systems Group to provide random samples of addresses. HINTS is approved by the Office of Management and Budget (approval number, 0925-0538), the office that reviews all federally sponsored surveys. Since the present study involves a secondary analysis of a publicly available dataset, it was exempt from institutional review board approval at the authors’ home institution. Full details about the HINTS methodology are available on the HINTS website [31].

### Study Design and Participants

The present study used a cross-sectional design to evaluate participant data from HINTS 5, Cycle 1 (N=3285). Survey responses were collected between January 25, 2017, and May 5, 2017, with complete data from 3191 respondents. The self-reported hypertension status was assessed using the question, “Has a doctor or other health professional ever told you that you had high blood pressure or hypertension (yes/no)?” Of the complete sample, approximately 1460 participants self-reported a history of hypertension (37.6% weighted prevalence).

### Measures

#### Mobile Device Ownership

Participants were asked if they owned a tablet computer like iPad, Samsung Galaxy, or Motorola Xoom; smartphone such as iPhone, Android, Blackberry, or Windows phone; or a basic mobile phone only. Response options were yes and no.

#### Apps Related to Health and Wellness

If participants responded “yes” to owning a tablet or smartphone, they were asked if they had any “apps” related to health and wellness, with response options of yes, no, and don’t know.

#### Tablet or Smartphone Helpfulness

Participants were asked if their tablet or smartphone ever helped them track progress on a health-related goal such as quitting smoking, losing weight, or increasing physical activity; make a decision about how to treat an illness or condition; and have discussions with their health care provider. The response options were yes and no.

#### Sociodemographic Factors

Covariates were selected based on known associations with hypertension, and mHealth broadly and included age, race/ethnicity, gender, and education [19,26,27,32,33]. Age was assessed as a categorical and continuous variable with age ranges 18-34 (reference), 35-49, 50-64, 65-74, and ≥75 years. Race/ethnicity was categorized into five categories: non-Hispanic white (reference), non-Hispanic black/African American, Hispanic, Non-Hispanic Asian, and non-Hispanic other. Gender was assessed as male (reference) and female. Education was classified into four categories: less than high school (reference), 12 years or completed high school, some college, and college degree. Income was not evaluated due to its collinearity with education.

### Statistical Analyses

Survey weights provided in the HINTS data were utilized to calculate weighted percentages of subject characteristics. To test for differences in outcomes across relevant patient characteristics, unadjusted chi-square tests were conducted on the weighted percent in agreement with various measures. Due to the survey weights, a Rao-Scott F-adjusted chi-square statistic was used, which yields a more conservative interpretation than the traditional Wald chi-square test used for unweighted analyses [34]. We used SAS software, version 9.4 of the SAS System, to conduct all analyses (SAS Institute Inc, Cary, NC), employing procedures that can account for the sampling design of HINTS, such as PROC SURVEYFREQ and PROC SURVEYREG. These procedures utilize the survey weights available in the HINTS data to obtain population-level point estimates and bootstrap accurate estimates of standard errors. To account for multiple comparisons, we applied methods to limit the false-discovery rate to 0.05 [35,36]. *P* values<.05 were considered statistically significant.

## Results

### Demographic Characteristics by Hypertension Status

Compared to participants without a self-reported history of hypertension, those with a history of hypertension were significantly more likely to be older, male, and black and have received less formal education (Table 1).

### Smartphone, Tablet, and Basic Mobile Phone Ownership by Hypertension Status

Of the full sample, approximately 62%, 79%, and 22% of HINTS participants reported owning a tablet, smartphone, and basic mobile phone only, respectively, and 84% reported that they had either a tablet or smartphone. When evaluating device ownership by hypertension status, fewer participants with a history of hypertension reported owning a tablet (55% vs 66%) or smartphone (68% vs 86%) than those without a history hypertension. Additionally, those with a self-reported history of hypertension were almost twice as likely to own a basic mobile phone only as compared to those without a history of hypertension (16.3% vs 8.5%).

**Table 1:** Participant characteristics according to the hypertension status.

Characteristics		All (N=3285), weighted %		Hypertension (N=1460), weighted % (95% CI)		Non-hypertension (N=1749), weighted % (95% CI)		*P* value	
**Age group (N=3095), years**									<.001
	18-34		21.96		5.11 (2.3-7.9)		31.97 (27.7-36.3)		
	35-49		28.77		22.05 (17.4-26.7)		32.76 (28.0-37.5)		
	50-64		30.25		39.89 (35.5-44.3)		24.52 (21.8-27.2)		
	65-74		10.92		17.13 (15.5-18.8)		7.23 (6.3-8.2)		
	≥75		8.1		15.82 (14.0-17.7)		3.52 (2.7-4.3)		
**Race/ethnicity (N=2906)**									<.001
	Non-Hispanic white		65.61		66.77 (63.2-70.3)		64.95 (62.9-67.0)		
	Non-Hispanic black		10.39		14.25 (12.0-16.5)		8.2 (6.7-9.7)		
	Hispanic		15.64		11.77 (9.3-14.3)		17.83 (16.2-19.4)		
	Non-Hispanic Asian		5.61		3.25 (1.7-4.8)		6.95 (5.9-8.0)		
	Non-Hispanic other		2.76		3.96 (2.8-5.1)		2.07 (1.5-2.7)		
**Gender (N=3161)**									0.03
	Male		49		52.6 (49.4-55.8)		46.85 (45.0-48.7)		
	Female		51		47.4 (44.2-50.6)		53.15 (51.3-55.0)		
**Education (N=3125)**									<.001
	Less than high school		8.6		11.28 (8.1-14.4)		6.99 (4.7-9.3)		
	High school		22.91		30.15 (25.7-34.6)		18.54 (15.9-21.2)		
	Some college		32.66		33.02 (29.0-37.0)		32.44 (29.9-35.0)		
	College degree or higher		35.83		25.55 (22.6-28.5)		42.02 (40.0-44.1)		
Have a tablet (N=3196)^a^		61.6		54.7 (50.7-58.8)		65.7 (61.0-70.3)		0.001	
Have a smartphone (N=3178)^a^		79		67.7 (64.1-71.4)		85.8 (82.9-88.6)		<.001	
Have a basic mobile phone (N=3124)^a^		21.5		28.9 (25.6-32.2)		17 (13.5-20.6)		<.001	
Have either a tablet or smartphone (N=3209)^a^		84.1		75.3 (71.6-79.0)		89.4 (87.1-91.7)		<.001	
Have a basic mobile phone, but neither a tablet nor a smartphone (N=3108)^a^		11.4		16.3 (13.4-19.2)		8.5 (6.4-10.5)		0.001	

^a^Values for these variables represent weighted percent of those who answered “Yes.”

### Smartphone, Tablet, and Basic Mobile Phone Ownership Among Participants With a History of Hypertension Only

There were significant differences in device ownership by age and education, but not race or gender (Tables 2 and 3). For example, participants aged 18-24 years were more likely to own a tablet or smartphone than those aged 65-74 years (90% vs 51% and 90% vs 61%, respectively; *P*<.001). Of the people aged ≥75 years, the majority owned a basic mobile phone only (47%) compared to a tablet (29%) or smartphone (30%). As the level of education of participants increased, so did the likelihood of owning a smartphone or tablet. Compared to participants with less than a high school education, those with a college degree or higher education were more likely to own a tablet and smartphone (41% vs 71% and 47% vs. 87%, respectively, *P*<.001).

**Table 2:** Differences in device ownership and use among patients with hypertension according to demographics. Sample sizes reflect the number of participants with nonmissing values for device question and characteristics.

Demographic		“Yes” to having a tablet (%)	“Yes” to having a smartphone (%)	“Yes” to having a mobile phone (%)	“Yes” to having a tablet or smartphone^a^ (%)	“Yes” to having a tablet and smartphone^b^ (%)
**Age group, years**		N=1389	N=1379	N=1350	N=1396	N=1377
	18-34	89.6	90.2	1.0	99.5	80.4
	35-49	61.1	86.7	15.5	93.9	46.1
	50-64	60.3	74.2	26.7	81.6	52.7
	65-74	51.4	60.9	40.4	68.2	43.3
	≥75	29.0	29.9	47.3	41.7	16.5
**Race/ethnicity**		N=1274	N=1267	N=1246	N=1277	N=1266
	Non-Hispanic white	58.1	71.7	26.9	79.5	50.0
	Non-Hispanic black	59.7	75.4	27.2	82.1	52.9
	Hispanic	48.9	74.8	24.5	80.1	43.3
	Non-Hispanic Asian	55.5	75.0	21.1	75.0	55.5
	Non-Hispanic other	78.8	60.2	44.5	83.4	55.7
**Gender**		N=1432	N=1419	N=1389	N=1440	N=1417
	Male	52.9	67.1	27.4	74.9	44.9
	Female	56.9	68.8	29.8	75.8	49.2
**Education**		N=1410	N=1398	N=1370	N=1417	N=1396
	Less than high school	40.7	46.5	29.5	52.8	34.3
	High school	44.9	53.4	43.6	66.4	31.1
	Some college	56.7	74.4	23.1	79.6	51.1
	College degree or higher	70.8	86.6	17.6	91.9	65.4

^a^Includes participants with nonmissing values for either device question (tablet or smartphone).

^b^Includes participants with nonmissing values for both device questions (tablet and smartphone).

**Table 3:** Statistical results of device ownership and use among patients with hypertension. Sample sizes reflect the number of participants with nonmissing values for device questions and characteristics.

Parameter		Wald chi-square value	F-adjusted chi-square value	*P* value
**Age group**				
	“Yes” to having a tablet (N=1389)	62.566	14.68	<.001
	“Yes” to having a smartphone (N=1379)	140.397	32.95	<.001
	“Yes” to having a mobile phone (N=1350)	52.49	12.32	<.001
	“Yes” to having a tablet or smartphone^a^ (N=1396)	121.111	28.42	<.001
	“Yes” to having a tablet and smartphone^b^ (N=1377)	75.7	17.77	<.001
**Race/ethnicity**				
	“Yes” to having a tablet (N=1274)	6.638	1.56	.20
	“Yes” to having a smartphone (N=1267)	1.69	0.40	.81
	“Yes” to having a mobile phone (N=1246)	3.29	0.77	.55
	“Yes” to having a tablet or smartphone^a^ (N=1277)	0.95	0.22	.92
	“Yes” to having a tablet and smartphone^b^ (N=1266)	1.57	0.369	.83
**Gender**				
	“Yes” to having a tablet (N=1432)	0.9871	—	.33
	“Yes” to having a smartphone (N=1419)	0.2128	—	.65
	“Yes” to having a mobile phone (N=1389)	0.5645	—	.46
	“Yes” to having a tablet or smartphone^a^ (N=1440)	0.0587	—	.81
	“Yes” to having a tablet and smartphone^b^ (N=1417)	1.134	—	.29
**Education**				
	“Yes” to having a tablet (N=1410)	55.912	17.88	<.001
	“Yes” to having a smartphone (N=1398)	64.93	20.76	<.001
	“Yes” to having a mobile phone (N=1370)	28.07	8.975	<.001
	“Yes” to having a tablet or smartphone^a^ (N=1417)	63.70	20.365	<.001
	“Yes” to having a tablet and smartphone^b^ (N=1396)	43.498	13.91	<.001

^a^Includes participants with nonmissing values for either device question (tablet or smartphone).

^b^Includes participants with nonmissing values for both device questions (tablet and smartphone).

### Presence of Health-Related Apps According to Hypertension Status

**Figure 1:**
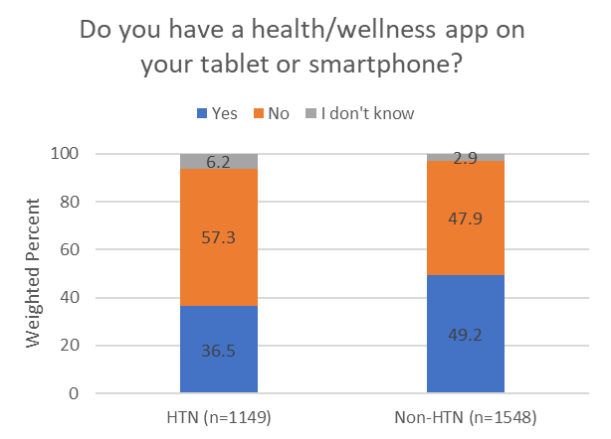
Use of health and wellness apps according to self-reported history of hypertension. Excludes those who answered “do not own a tablet or smartphone”. HTN: hypertension.

Significant differences were observed between participants with and those without a self-reported history of hypertension with regard to having health-related apps on their tablet or smartphone. For example, only 36.5% of participants with a history of hypertension reported having health-related apps compared to 49.2% of those without a history of hypertension (Figure 1; *P*<.001). Participants with a self-reported history of hypertension were also more likely to report that they did not know if they had a health-related app compared to those without a history of hypertension (ie, 6.2% vs 2.9%).

**Figure 2:**
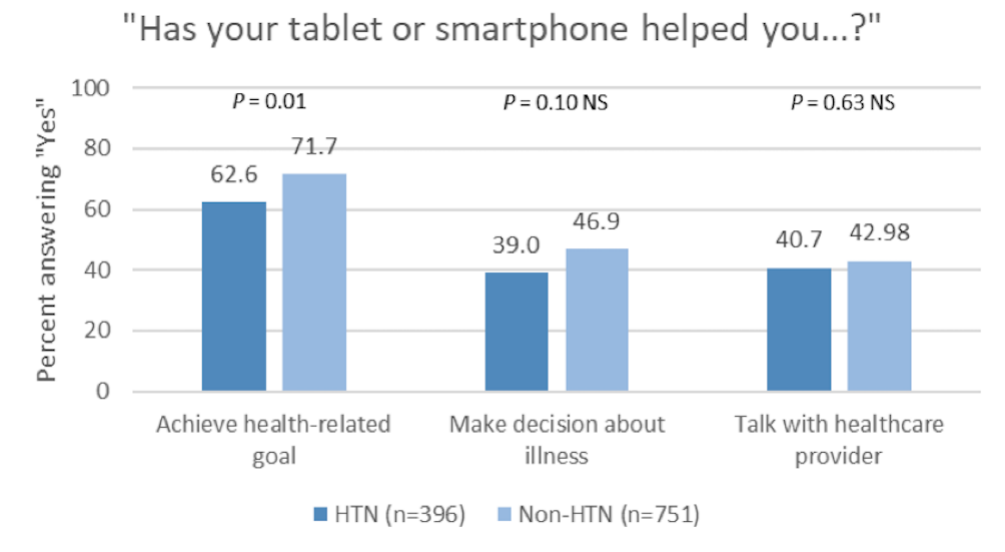
How tablets and smartphones help people reach goals, make decisions, and talk to health care providers according to self-reported history of hypertension. Excludes those who answered “do not own a tablet or smartphone." HTN: hypertension.

Among participants who owned a tablet or smartphone (Figure 2), those with a self-reported history of hypertension were less likely to report that their tablet or smartphone helped them reached a health-related goal like quitting smoking, losing weight, or increasing physical activity (62.6% vs 71.7%, *P*=.02). With regard to whether tablets or smartphones helped people make a decision about an illness or talk to health care providers, no differences were observed between participants with and those without a history of hypertension.

### Presence of Health-Related Apps According to Sociodemographics Among Participants With a History of Hypertension Only

**Figure 3:**
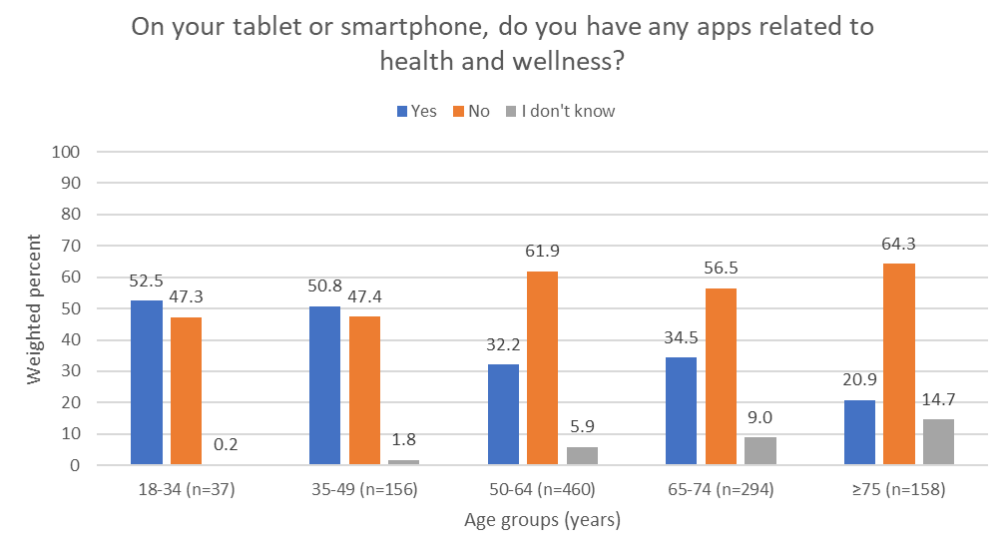
Among those with a self-reported history of hypertension exclusively, differences in health/wellness app use according to age. P=.001 for the test of whether the distribution of responses (“yes”, “no”, or “I don’t know”) differs by age group. Excludes those who answered “do not own a tablet or smartphone.”.

General trends were observed with regard to age when examining HINTS participants with a self-reported history of hypertension exclusively (Figure 3). Participants with health-related apps on their tablets or smartphones were mostly in age categories of 18-34 years (53%) and 35-49 years (51%), whereas percentages for age groups of 50-64, 65-74, and ≥75 years were lower (32%, 35%, and 21%, respectively). Participants who were ≥50 years of age were more likely to report that they did not know if they had any health-related apps on their tablets and smartphones.

## Discussion

### Principal Findings

The purpose of this study was to describe the prevalence of mobile device ownership and presence of health-related apps according to the self-reported hypertension status and sociodemographic factors in a nationally representative sample of US adults. We also examined whether smartphones and tablets helped people improve health goal achievement, medical decision making, and patient-provider communication. In summary, we found that people with a self-reported history of hypertension were less likely to own a smartphone or tablet, have health apps on their mobile devices, and report that smartphone and tablets helped them achieve a health-related goal compared to those without a self-reported history of hypertension. We also found that among people with a history of hypertension exclusively, smartphone ownership was associated with age and education, but not race or sex. Specifically, younger people and people with a college education are more likely to have smartphones, whereas older adults and people with a less formal education are more likely to have basic mobile phones.

A key finding from our study was that participants with a self-reported history of hypertension were significantly less likely to have health-related apps on their smartphones or tablets than those without a self-reported history of hypertension. This finding differs from other research that has shown no difference in health app downloads between people with and those without a chronic illness [18]. It is possible that people without a self-reported history of hypertension had other medical conditions for which they were using health-related apps (eg, type 2 diabetes) or they used general health-promotion apps more often (eg, apps to help track diet and physical activity). Researchers should be cautious about solely relying on health apps to support hypertension self-management by patients, as such reliance may cause them to overlook patients who do not use health apps. It is also possible that some patients were not using health-related apps due to limited eHealth literacy [37], attitudes about technology [38], or challenges with self-regulation and self-control [39,40]. Nevertheless, health-related apps may still be promising tools for a subset of the population that likes to engage with mHealth tools and prefers technology-based strategies for managing health.

Regarding the various ways that smartphones or tablets may help people, participants with a self-reported history of hypertension were less likely to report that their smartphones and tablets helped them reach a health-related goal like quitting smoking, losing weight, or increasing physical activity. This finding is notable, given that lifestyle behaviors can help people manage hypertension [41] and that tracking behaviors is a common feature of many health apps [42].

### Strengths and Limitations

A strength of this study was our use of the HINTS data set. HINTS collects nationally representative data every few years from a large sample of US adults. This routine data collection allows researchers to monitor health communication trends over time. Given that many survey items are unique to HINTS and not available in other publicly available data sets, HINTS is a rich resource for evaluating various aspects of health behavior, information seeking, and trends in health communication. Despite its strengths, some limitations of this study must be noted. The hypertension status was self-reported in HINTS and not confirmed through a clinical diagnosis. Over- or underreporting of hypertension may have affected the results in either direction; however, we are unable to quantify the degree of over- or underreporting in the HINTS data set. We have no information about whether people who took the survey were currently receiving antihypertensive medication; the level of blood pressure control among medication users, which may have implications for health app use generally; and how people may use mobile devices for health and wellness. We are unable to distinguish between the various types of hypertension that a person may have experienced (eg, pregnancy related, primary hypertension, or secondary hypertension caused by another medical problem). We do not have information about when participants were ever informed that they had hypertension. It is possible that people who were recently informed (eg, within the last 12 months) would be more motivated to use health-related apps than people who have been living with hypertension for many years, or vice versa.

### Future Directions for Research

Given the small but growing amount of data on mHealth interventions for hypertension, our findings raise several questions for future investigation. For example, because there are many ways to support hypertension prevention and management (eg, pharmacological, nonpharmacological, mHealth, eHealth, print, and in person), more research is needed to determine patients’ preferences for various interventions and whether these preferences are associated with long-term engagement. For example, it is possible that some people may prefer telephone-based counseling or text messaging interventions over health-related apps when given the choice. Further evaluation of which behavioral theory (or combination of theories) best predicts hypertension-related mHealth intervention uptake is also needed. A recent systematic review noted that few mHealth interventions are based on behavioral theories [43]. Of the limited studies that highlight such theories, the Health Belief Model is commonly selected [43]. Moreover, as more health apps are developed, further examination of the features that matter most to patients and the quality of these components warrant more attention [44,45]. For example, Khalid et al found that privacy and ethics concerns, hidden costs, interface design, and app crashes were commonly reported complaints among mobile app users [46]. Finally, further research on the correlations between an individual’s eHealth literacy and mHealth use may inform how patients will respond to mHealth interventions for hypertension in the future [47].

### Conclusions

Smartphone and tablet ownership and the presence of health apps on mobile devices are less common in people with a self-reported history of hypertension compared to those without a history of hypertension. Future studies should examine how to disseminate and implement mHealth interventions in the populations most affected by hypertension. Moving forward, a combination of novel mHealth interventions and traditional health communication strategies (eg, print, web based, in person, and telephone based) may be needed to reach a wide cross-section of patients with a self-reported history of hypertension.

## Abbreviations

eHealth: electronic health
HINTS: Health Information National Trends Survey
mHealth: mobile health
